# Biosynthetic Pathways and Regulation of Antibacterial Compounds From Endophytic Fungi: From Chemistry to Genomics and Biotechnology

**DOI:** 10.1002/cbdv.71888

**Published:** 2026-10-30

**Authors:** Farkhod Eshboev, Abubakar Siddiq Salihu, Lobar Khayrullaeva, Doniyor Khamdamov, Alex X. Gao, Kamila Mardieva, Wan Mohd Nuzul Hakimi Wan Salleh, Shakhnoz Azimova, Karl W. K. Tsim

**Affiliations:** ^1^ Institute For Advanced Studies New Uzbekistan University Tashkent Uzbekistan; ^2^ Department of Chemistry, Faculty of Science and Mathematics Universiti Pendidikan Sultan Idris Tanjong Malim Perak Malaysia; ^3^ Department of Pure and Industrial Chemistry, Faculty of Natural and Applied Sciences Umaru Musa Yar'adua University Katsina Nigeria; ^4^ Faculty of Chemistry and Biology Karshi State University Karshi Uzbekistan; ^5^ Division of Life Science and Center for Chinese Medicine R&D The Hong Kong University of Science and Technology Clear Water Bay Kowloon Hong Kong SAR China; ^6^ S.Yu. Yunusov Institute of the Chemistry of Plant Substances Academy of Sciences of Uzbekistan Tashkent Uzbekistan

**Keywords:** antibacterial compounds, antimicrobial resistance, biosynthetic gene clusters, endophytic fungi, genome mining

## Abstract

Endophytic fungi are non‐pathogenic symbionts that represent a rich source of structurally diverse bioactive secondary metabolites. However, genome‐mining studies reveal that most of their biosynthetic gene clusters (BGCs) remain transcriptionally silent under standard laboratory conditions, leaving much of their chemical diversity unexplored. This review examines antibacterial natural products from endophytic fungi by integrating three complementary aspects: metabolite chemistry, biosynthetic pathways, and strategies for activating cryptic BGCs. Endophytic fungi produce polyketides, non‐ribosomal peptides, terpenoids, phenylpropanoid‐derived compounds, and hybrid metabolites with antibacterial activities mediated through inhibition of ribosomes, cell wall and membrane biosynthesis, and bacterial resistance mechanisms. Their genomes encode diverse PKS, NRPS, TPS, and hybrid biosynthetic systems, many of which remain cryptic. Advances in chemical elicitation, co‐cultivation, epigenetic modulation, CRISPR‐based engineering, genome mining, and machine learning are accelerating the activation of silent clusters and the assignment of BGCs to their metabolites. Several fungal metabolites exhibit potent activity against multidrug‐resistant pathogens, including MRSA, and some enhance the efficacy of existing antibiotics. This review highlights current progress, identifies remaining challenges in metabolite production and BGC annotation, and outlines future directions for translating endophytic fungi into a sustainable platform for next‐generation antibacterial drug discovery.

## Graphical abstract

Endophytic fungi encode abundant cryptic biosynthetic gene clusters that remain largely silent under conventional cultivation. This review highlights how genome mining, multi‐omics, and cluster activation strategies bridge the BGC‐to‐metabolite gap, enabling the discovery of structurally diverse antibacterial natural products with activity against multidrug‐resistant pathogens

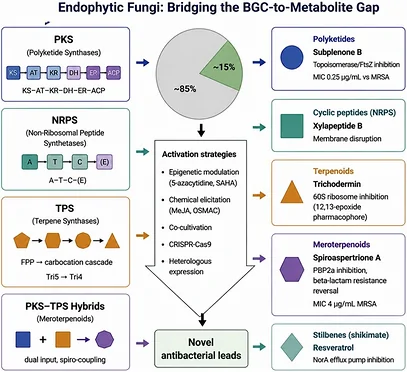

## 1 Introduction

Antimicrobial resistance (AMR) has emerged as one of the most critical public health crises of the 21st century. A global burden‐of‐disease analysis published in *The Lancet* estimated that bacterial AMR was directly responsible for 1.27 million deaths in 2019 and contributed to approximately 4.95 million deaths globally, a toll that exceeded those of HIV/AIDS and malaria in the same period [1]. The World Health Organization projects that, absent coordinated intervention, drug‐resistant infections could account for up to 10 million deaths annually by 2050 [2]. A particularly alarming feature of this crisis is the near‐total absence of novel antibiotic classes entering clinical use: the last structurally distinct mechanistic class to achieve approval was the lipopeptide daptomycin in 2003, and no compound with a genuinely new bacterial target mechanism has advanced through Phase III trials since that time. This stagnation reflects both the biological difficulty of identifying compounds active against established resistance mechanisms and the well‐documented commercial disincentives that have driven pharmaceutical investment away from anti‐infective development [3].

Natural products continue to serve as the primary chemical source of antibacterial agents, with most antibiotics currently in clinical use or late‐stage development originating from natural compounds or their semi‐synthetic derivatives [4]. Fungi have been particularly productive in this regard, having provided penicillin, cephalosporins, fusidic acid, pleuromutilin, and griseofulvin, among others. Endophytic fungi represent a distinct and still largely underexplored subset of the fungal kingdom. Defined as microorganisms that colonize internal plant tissues without causing visible pathological symptoms [5], endophytes occupy a nutritionally and ecologically complex niche that appears to select for chemical diversity. Their secondary metabolites are thought to serve multiple ecological functions: defense against competing microorganisms within the same host tissue, mediation of host plant immunity, and responses to abiotic stress signals from the plant. This ecological context, where endophytes face constant competition and chemical signaling from a structurally diverse plant microbiome, may explain why their secondary metabolomes span an unusually wide range of structural classes and mechanisms of action [6, 7].

The first indication that endophytic chemistry exceeded what classical isolation could reveal came from early reports that metabolites long considered exclusive plant products, including paclitaxel, camptothecin, and podophyllotoxin, could be isolated from endophytic fungal partners of the same host plants [6, 8]. Whether these observations reflect horizontal gene transfer, convergent evolution, or methodological contamination has remained a point of genuine scientific debate, but the discoveries established that endophytic biosynthetic capacity was more extensive than anticipated. The genomic dimension of this story became fully apparent with the widespread adoption of whole‐genome sequencing and automated BGC detection software such as antiSMASH (Antibiotics and Secondary Metabolite Analysis Shell). Analysis of endophytic fungal genomes routinely identifies 30 to 80 BGCs per isolate, yet metabolite profiling under standard culture conditions rarely detects products corresponding to more than 10 to 20 percent of these clusters [9, 10, 11]. The remainder are classified as silent or cryptic: their genes are present and structurally intact but are not transcribed at detectable levels under typical laboratory conditions. This genomic evidence establishes that the chemical potential of endophytic fungi has been systematically underestimated, and that the gap between genomic capacity and observed output is not a minor discrepancy but a fundamental limitation of current discovery approaches.

Understanding why BGCs are silent and how they can be activated requires mechanistic knowledge at multiple levels. At the chromatin level, silent clusters are frequently maintained in heterochromatic states through histone H3 lysine 9 trimethylation (H3K9me3) and reduced histone acetylation, enforced by the heterochromatin protein HepA and maintained through the activity of Hda1 and Rpd3 family histone deacetylases [12, 13]. At the signaling level, cluster activation is linked to environmental cues that funnel through conserved fungal regulatory networks involving the velvet complex (VeA, VelB, and LaeA) and cAMP‐protein kinase A cascades [14, 15]. At the ecological level, the silence of most BGCs under monoculture likely reflects the absence of the competitive or cooperative signals that normally trigger their expression in planta or in mixed‐species communities. These regulatory layers are not independent problems; activating silent clusters systematically requires an understanding of all three.

Against this background, the present review has three specific objectives. First, it examines the structural chemistry and mechanisms of action of the major classes of antibacterial compounds reported from endophytic fungi, with emphasis on the relationship between chemical structure and antibacterial target. Second, it analyzes the biosynthetic pathways responsible for producing these compound classes, identifying the enzymatic logic that generates structural complexity and the regulatory architecture that controls pathway expression. Third, it critically evaluates the strategies currently used to activate silent clusters and improve metabolite yields, assessing the mechanistic basis, demonstrated successes, and current limitations of each approach. The review concludes by proposing a prioritized research framework that links genomic activation, mechanistic chemistry, and translational biology as an integrated path toward realizing the antibacterial potential of endophytic fungi.

## 2 Chemical Diversity and Mechanisms of Action of Antibacterial Compounds From Endophytic Fungi

Endophytic fungi produce structurally diverse antibacterial metabolites across all major secondary metabolite classes, including polyketides, terpenoids, non‐ribosomal peptides, phenylpropanoid‐derived compounds, and complex hybrid molecules. This chemical diversity reflects the ecological pressure these organisms face within host plant tissues, where competition with bacteria, other fungi, and the plant immune system selects for metabolites with diverse modes of action. A critical feature that distinguishes endophytic chemistry from that of soil fungi or marine organisms is the frequent production of compounds with mechanisms that go beyond simple growth inhibition: several endophytic metabolites act as resistance‐pathway modulators, restoring antibiotic susceptibility in drug‐resistant pathogens by targeting efflux pumps, penicillin‐binding proteins, or membrane composition [4, 16]. Evidence supporting this broader mechanistic profile includes: spiroaspertrione A restores oxacillin efficacy against MRSA via PBP2a inhibition [16], resveratrol inhibits NorA‐type efflux pumps [29], and phomodione dissipates the bacterial proton motive force [30]. Understanding the antibacterial mechanisms of these compound classes is essential for evaluating their translational potential.

### 2.1 Polyketides: Structural Diversity and DNA/Enzyme‐Targeting Mechanisms

Fungal polyketides are assembled by iterative type I polyketide synthases (iPKSs) that extend a starter unit through repeated Claisen condensation of malonyl‐CoA extender units, with optional reductive processing at each cycle. The degree of reduction, the choice of starter unit, and post‐assembly tailoring reactions including hydroxylation, methylation, glycosylation, and oxidative cyclization collectively determine whether the final product adopts a phenolic, quinone, xanthone, macrolide, or chromene scaffold [17]. This enzymatic flexibility accounts for the exceptional structural range observed in endophytic polyketides and, directly, for the range of bacterial targets they engage.

Penialidin C, isolated from *Penicillium* sp. associated with *Garcinia nobilis* leaves, inhibits *S. aureus* and *Bacillus subtilis* at MIC 5 µg/mL, comparable to streptomycin [18]. Its pentalene‐fused lactone core is structurally analogous to known DNA gyrase‐targeting agents, suggesting topoisomerase interference as a probable mechanism. The dimeric xanthones subplenones A–J from *Subplenodomus* sp. is more instructive: their biaryl ether linkage and hydroxyl‐substituted aromatic rings are features shared with xanthone‐class gyrase poisons, and subplenone B inhibits MRSA at MIC 0.25 µg/mL [19], a potency consistent with topoisomerase or FtsZ interference rather than non‐specific membrane disruption. The macrodiolide parengyomarins A–B from the mangrove endophyte *Parengyodontium album* (MIC 0.4 µM against *S. aureus*) exemplify a macrolide membrane‐insertion strategy [20]. From a structure–activity perspective, the antibacterial potency of endophytic polyketides correlates with aromatic ring oxidation state, hydrogen‐bond donor–acceptor geometry, and macrocyclization. Planar, electron‐rich aromatic systems with free hydroxyl groups favor intercalative or topoisomerase‐targeting mechanisms; macrolides favor membrane disruption or ribosome interaction depending on ring size; linear polyketides show weaker activity unless halogen substituents enhance membrane partitioning.

### 2.2 Terpenoids: Membrane Disruption, Ribosome Inhibition, and Sterol Pathway Interference

Terpenoids from endophytic fungi span sesquiterpenes (C15), diterpenes (C20), and triterpenes (C30), each class carrying distinct structural and mechanistic properties. Cadinane‐type sesquiterpenoids from *Trichoderma virens* (an endophyte of *Artemisia argyi*) inhibit Gram‐negative bacteria and plant‐pathogenic fungi [21], likely through disruption of bacterial cell membranes: the compact hydrophobic ring system of cadinane sesquiterpenes partitions into lipid bilayers, increasing membrane fluidity and permeability at sub‐lethal concentrations with bactericidal activity at higher doses. Trichothecene sesquiterpenes, including trichodermin produced by Trichoderma endophytes through the tri gene cluster, inhibit eukaryotic ribosomal peptidyl transferase activity by binding within the A‐site cleft of the 60S ribosomal subunit, blocking peptide bond formation [22, 23]. In vitro anti‐MRSA activity has been documented for trichodermin [23], the molecular basis of this antibacterial activity is distinct from eukaryotic ribosome inhibition since prokaryotic 70S ribosomes differ structurally at the peptidyl transferase center, and likely involves a secondary mechanism at higher concentrations. Helvolic acid, a fusidane‐type triterpene from *Aspergillus* endophytes, shares structural features with fusidic acid, a clinical antibiotic that traps the elongation factor G (EF‐G):GDP:ribosome ternary complex in the post‐translocational state, blocking protein synthesis [24]. The independent production of a fusidane scaffold by endophytic fungi strongly implies that this chemical architecture has been selected for antibacterial activity across independent evolutionary lineages.

### 2.3 Non‐Ribosomal Peptides: Membrane Disruption, Ionophore Activity, and Enzyme Inhibition

Non‐ribosomal peptides (NRPs) assembled by NRPS megaenzymes contain non‐proteinogenic residues that impose conformational constraints not achievable through ribosomal synthesis. Xylapeptide B, a cyclic pentapeptide from a Xylaria endophyte of *Sophora tonkinensis*, contains l‐pipecolinic acid, a cyclic amino acid that restricts backbone conformation and creates a well‐defined hydrophobic face on the macrocycle [25]. This amphipathic topology is characteristic of membrane‐active peptides that disrupt membrane integrity through pore formation or bilayer thinning, and is consistent with the broad‐spectrum activity against Gram‐positive bacteria and *Candida albicans* at low micromolar concentrations. Beauvericin, a cyclic depsipeptide produced by *Fusarium oxysporum* endophytes, is an ionophore that forms cation‐selective channels across biological membranes by complexing monovalent cations (K^+^, Na^+^) and shuttling them down electrochemical gradients, dissipating membrane potential [26, 27]. The alternating N‐methyl‐l‐phenylalanine and d‐hydroxyisovalerate residues in the hexadepsipeptide ring allow it to adopt lipophilic conformations for membrane insertion and polar conformations for ion coordination. Resistance to ionophore mechanisms is difficult to develop through single‐target mutation because the target is the biophysical property of membrane potential rather than a specific protein.

### 2.4 Phenylpropanoid and Flavonoid‐Type Compounds: Cell Wall, Efflux, and Energy Coupling

Phenylpropanoid and xanthone‐type compounds from endophytic fungi engage bacterial targets through multiple routes. Xanthoascin from *Aspergillus* sp. IFB‐YXS shows potency against *Clavibacter michiganensis* exceeding that of streptomycin [28]; its isocyanide‐related functional group is a known pharmacophore for metalloenzyme inhibition, particularly of zinc‐containing metalloproteinases. Resveratrol, produced by *Alternaria* sp. MG1 through a PAL‐4CL pathway [29], has been shown to inhibit FtsZ polymerization in bacterial cell division and to function as an efflux pump inhibitor (EPI) by occupying the substrate‐binding pocket of NorA‐type multidrug efflux proteins in S. aureus. The EPI activity of resveratrol is particularly relevant to AMR because it does not require intrinsic bactericidal potency; it restores susceptibility of efflux‐overexpressing strains to fluoroquinolones and tetracyclines by blocking their export. Phomodione, a furandione related to usnic acid from *Phoma* sp. [30], is a structural analogue of known uncouplers of bacterial oxidative phosphorylation that transport protons across the inner membrane and dissipate the proton motive force required for ATP synthesis, a mechanism that affects any bacterium relying on oxidative phosphorylation and is not readily evaded through single‐gene mutation.

### 2.5 Hybrid Metabolites: Resistance Modulation as a Distinct Antibacterial Strategy

Meroterpenoids and PKS‐NRPS hybrids from endophytic fungi include some of the most pharmacologically significant compounds documented in this review because several exploit a qualitatively different antibacterial strategy: disabling resistance mechanisms rather than killing bacteria directly. *Aspergillus* sp. TJ23, an endophyte of *Hypericum perforatum*, produces spiroaspertrione A, a spirocyclic meroterpenoid that inhibits MRSA at MIC 4 µg/mL and, more significantly, reduces the oxacillin MIC against MRSA from above 128 µg/mL to below 2 µg/mL [16]. This beta‐lactam‐potentiating activity most probably reflects inhibition of penicillin‐binding protein 2a (PBP2a), the product of the mecA gene that confers methicillin resistance. Aspermerodione, also from *Aspergillus* sp. TJ23 and carrying an unusual 2,6‐dioxabicyclo[2.2.1] heptane skeleton, potentiates beta‐lactams through a structurally distinct PBP2a interaction, [31] establishing that a single endophyte has independently evolved at least two chemical solutions to the same resistance target. Cytochalasins, macrocyclic PKS‐NRPS hybrids from *Xylaria*, *Diaporthe*, and *Phomopsis* endophytes, engage FtsZ, the bacterial tubulin homologue forming the Z‐ring essential for cytokinesis, through their rigid macrocyclic architecture [32, 33]. FtsZ has no mammalian homologue, making cytochalasin scaffolds potentially selective antibacterial leads if the cytotoxic actin‐binding activity can be structurally uncoupled from FtsZ inhibition. Table 1 summarizes the structural class, producing organism, host plant, bioactivity, and proposed primary antibacterial mechanism for representative compounds discussed in this section.

**TABLE 1:** Representative antibacterial compounds from endophytic fungi with proposed primary mechanisms.

Compound	Class	Endophyte	Host plant	Activity (MIC)	Proposed mechanism	Ref.
Penialidin C	Polyketide (pentalene lactone)	*Penicillium sp*.	*Garcinia leaves*	MIC 5 µg/mL (S. aureus, B. subtilis)	DNA gyrase inhibition (putative*)	[18]
Subplenones A–J	PKS xanthone (dimeric)	*Subplenodomus sp*.	*Gentiana roots*	MIC 0.25 µg/mL (MRSA)	Topoisomerase/FtsZ inhibition	[19]
Parengyomarins A–B	PKS macrodiolide	*Parengyodontium album*	*Avicennia marina*	MIC 0.4 µM (S. aureus)	Membrane insertion (macrocycle)	[20]
Cadinane sesquiterpenoids	Sesquiterpene	*Trichoderma virens*	*Artemisia argyi*	Broad spectrum (MIC ND)	Membrane fluidity disruption	[21]
Harziane diterpenoids	Diterpene	*Trichoderma sp*.	*Xylaria*	MIC < 10 µg/mL (S. aureus)	Membrane interference (lipophilic)	[21]
Trichodermin	Trichothecene sesquiterpene	*Trichoderma sp*.	*Various*	Anti‐MRSA (in vitro)	Ribosomal peptidyl transferase inhibition (eukaryotic 60S); secondary mechanism vs. bacteria	[23]
Helvolic acid	Fusidane triterpene	*Aspergillus sp*.	*Various*	Antibacterial	EF‐G:GDP:ribosome complex trapping	[24]
Xylapeptide B	Cyclic pentapeptide (NRPS)	*Xylaria sp*.	*Sophora tonkinensis*	Low µM (Gram+)	Amphipathic membrane disruption	[25]
Beauvericin	Cyclic depsipeptide	*Fusarium oxysporum*	*Edgeworthia*	MIC 3.9 µM (S. aureus)	Ionophore; membrane potential dissipation	[26]
Xanthoascin	Phenolic xanthone	*Aspergillus sp. IFB‐YXS*	*Ginkgo biloba*	Exceeds streptomycin potency	Metalloenzyme inhibition (isocyanide)	[28]
Resveratrol	Stilbene (shikimate)	*Alternaria sp. MG1*	*Grapevine*	Phytoalexin range	FtsZ inhibition; NorA efflux pump inhibition	[29]
Phomodione	Furandione (phenolic)	*Phoma sp*.	*Saurauia scaberrinae*	Anti‐S. aureus	Proton motive force uncoupling	[30]
Spiroaspertrione A	Meroterpenoid	*Aspergillus sp. TJ23*	*Hypericum perforatum*	MIC 4 µg/mL MRSA; potentiates oxacillin	PBP2a inhibition; beta‐lactam resistance reversal	[16]
Aspermerodione	Meroterpenoid (bicyclic)	*Aspergillus sp. TJ23*	*Hypericum perforatum*	Beta‐lactam potentiation	PBP2a active site inhibition	[31]
Cytochalasins	PKS‐NRPS macrocycle	*Xylaria sp. / Phomopsis sp*.	*Kandelia candel*	MRSA inhibition (µM)	FtsZ polymerization inhibition; actin disruption	[32, 33]

*Mechanisms labelled “putative” are inferred from structural analogy to known pharmacophores and have not been confirmed by direct biochemical assay for the specific compound listed.

## 3 Biosynthetic Pathways: Enzymatic Logic, Structural Outcomes, and Regulatory Architecture

The chemical diversity described in Section 2 is generated by five major biosynthetic systems: polyketide synthases (PKS), non‐ribosomal peptide synthetases (NRPS), hybrid PKS‐NRPS assemblies, terpene synthases (TPS), and shikimate‐phenylpropanoid enzymes (Figure 1). This section examines the enzymatic mechanisms that produce structural complexity within each pathway family and the domain‐level logic that explains how small sets of genes generate large structural diversity. Understanding this biosynthetic logic is a prerequisite for rational genome mining, without knowing how PKS domain combinations generate different carbon backbone geometries, or why NRPS adenylation domain specificity codes can tolerate non‐natural substrates, researchers cannot make meaningful predictions about the structures encoded by orphan clusters.

**FIGURE 1:**
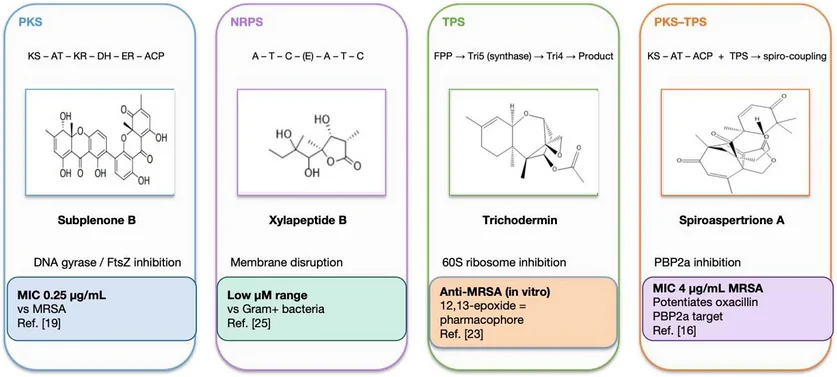
Major biosynthetic pathway families in endophytic fungi and representative antibacterial compounds.

### 3.1 Polyketide Synthases: Iterative Programming and Structural Diversification

Fungal PKSs are iterative type I enzymes, meaning that a single set of catalytic domains performs multiple rounds of chain extension on the same growing intermediate before product release. This architecture differs fundamentally from the assembly‐line (modular) PKSs of bacteria, where each domain acts only once. In fungal iPKSs, the KS (ketosynthase), AT (acyltransferase), and ACP (acyl carrier protein) domains are obligatory and catalyze condensation. Optional reductive domains, KR (ketoreductase), DH (dehydratase), and ER (enoylreductase), are applied selectively at each cycle according to a programming rule encoded in the KS domain itself. The result is a controlled pattern of keto, hydroxyl, olefinic, and methylene functionalities along the carbon backbone that determines the final cyclization mode and scaffold class [17].

Highly reducing PKSs (HR‐PKS) apply all three reductive domains in most cycles, producing reduced chains that cyclize into decalin, naphthalene, or chromene frameworks. Non‐reducing PKSs (NR‐PKS) apply no reductive steps, producing fully aromatic polyketide chains that cyclize into phenolic structures such as anthraquinones and xanthones. The subplenone xanthones [19], with their highly conjugated biaryl ether systems and potent MRSA activity, are NR‐PKS products; their structural planarity and electron density directly result from the absence of reductive processing during chain assembly. Post‐assembly tailoring by cytochrome P450 monooxygenases, flavin‐dependent oxidases, and O‐methyltransferases introduces the hydroxyl, epoxide, methoxy, and carboxyl groups that fine‐tune bioactivity and solubility. The remarkable potency of parengyomarin macrodiolides [20] against *S. aureus* is attributable not just to macrolide ring size but to the specific configuration of hydroxyl groups introduced by CYP tailoring enzymes that orient the molecule for membrane insertion.

### 3.2 Non‐Ribosomal Peptide Synthetases: Module Logic and Non‐Proteinogenic Residues

NRPS enzymes operate as modular assembly lines in which each module incorporates one amino acid into the growing peptide chain through the sequential action of adenylation (A), thiolation (T), and condensation (C) domains. Epimerization (E) domains convert l‐configured amino acids to d‐forms in situ, generating d‐residues that confer conformational rigidity and protease resistance to the final peptide [34]. The antibacterial selectivity and potency of NRPS products depend critically on the non‐proteinogenic residues incorporated. Xylapeptide B contains l‐pipecolinic acid, a six‐membered ring analogue of proline that dramatically restricts backbone flexibility and enforces the amphipathic conformation required for membrane insertion [25]. Beauvericin incorporates N‐methyl‐l‐phenylalanine and d‐hydroxyisovalerate; N‐methylation increases lipophilicity and membrane partitioning, while d‐amino acid configuration allows the macrocycle to adopt the bowl‐like conformation required for ion coordination [26]. The substrate specificity of A domains is determined by a 10‐residue non‐ribosomal code in the binding pocket, and predicting this specificity from genome sequence using tools such as NRPSpredictor2 and SANDPUMA achieves around 70%–80% accuracy [35], making NRPS clusters tractable targets for structure prediction from BGC sequences, though accuracy decreases for rare or novel substrates.

### 3.3 PKS‐NRPS Hybrid Pathways: Expanding Chemical Space Through Cross‐Domain Assembly

Hybrid PKS‐NRPS pathways generate compounds whose structural complexity exceeds what either pathway alone can produce. The junction between polyketide and peptide assembly is mediated by docking domains or CoA‐ligase‐like activities that activate the polyketide terminus for NRPS loading. Cytochalasins are the most extensively characterized endophytic PKS‐NRPS hybrids: their biosynthesis begins with a PKS module that builds a reduced polyketide chain, which is then loaded onto an NRPS module that incorporates a single amino acid (typically phenylalanine or tryptophan) and releases the product by intramolecular Diels–Alder cyclization, generating the rigid macrocyclic carbocycle‐fused ring system that confers both actin capping and FtsZ inhibitory activities [32, 33]. The fact that both activities reside in the same scaffold has historically limited cytochalasin development, but structure‐guided modifications targeting the actin‐binding interface while preserving FtsZ contacts represent a tractable medicinal chemistry opportunity. Fusaristatin A is a PKS‐NRPS lipopeptide where a PKS‐assembled fatty acid chain is extended by NRPS modules to produce an antibacterial acylpeptide [36], and beauvericin, though primarily NRPS‐assembled, uses a PKS‐derived hydroxy acid extender, placing it at the boundary of the two pathway types [26, 27].

### 3.4 Terpene Synthases: Carbocation Cascades and Structural Rigidity

Terpene biosynthesis in fungi begins with the mevalonate pathway, which produces farnesyl diphosphate (FPP, C15) and geranylgeranyl diphosphate (GGPP, C20) as the direct substrates for sesquiterpene and diterpene synthases, respectively. The synthase initiates an electrophilic cascade by ionizing the diphosphate, generating a carbocation that undergoes sequential cyclizations, hydride shifts, and methyl migrations before being quenched by deprotonation or water capture. The trichothecene pathway producing trichodermin in *Trichoderma* endophytes exemplifies pathway‐level complexity: tri5 (trichodiene synthase) cyclizes FPP to trichodiene, and subsequent oxidations by the multifunctional CYP450 tri4, followed by acetylations and isomerizations by other cluster enzymes, convert trichodiene to the final trichothecene product [22, 23]. The 12,13‐epoxide group introduced by tri4 is the pharmacophore that confers ribosome‐inhibitory activity: removal of this epoxide abolishes antifungal activity, demonstrating that a single CYP450 tailoring step is the pharmacophore‐generating event. Helvolic acid biosynthesis involves a triterpene cyclization pathway that produces a fusidane skeleton through an unusual C‐4 demethylation process distinct from sterol biosynthesis [24].

### 3.5 Shikimate and Phenylpropanoid Pathways: Evidence, Controversy, and Biosynthetic Implications

The presence of canonical plant‐type shikimate pathway enzymes, including phenylalanine ammonia lyase (PAL), 4‐coumarate:CoA ligase (4CL), and chalcone synthase‐like type III PKSs, in endophytic fungi has been documented transcriptomically in *Alternaria* sp. MG1 [29], *Eupenicillium* sp. [37], and *Thielavia* endophytes of *Hypericum* species, among others. Whether these observations reflect horizontal gene transfer from the host plant, convergent evolution of parallel enzymatic routes, or systematic laboratory contamination during isolation has not been definitively resolved. The deduced amino acid sequences share 45%–65% identity with plant homologues but are phylogenetically distinct from fungal fatty acid synthases, making de novo evolution less parsimonious than horizontal acquisition from the host. However, rigorous chromosomal localization of these genes in long‐read assemblies and comprehensive phylogenomic analysis of plant‐endophyte gene tree topologies would be needed to distinguish these hypotheses convincingly. The practical implication for genome mining is that standard fungal BGC detection pipelines trained on canonical fungal biosynthetic signatures will miss these plant‐like clusters, representing a systematic blind spot in current BGC inventories.

Ribosomally synthesized and post‐translationally modified peptides (RiPPs) represent a growing class of fungal secondary metabolites that warrants acknowledgment, though they are less extensively characterized in endophytic fungi than in bacteria. Fungal RiPPs, including ustiloxins, phomopsins, and borosins, are produced by ribosomal synthesis of a precursor peptide followed by extensive post‐translational modifications such as cyclization, methylation, and macrolactamization, catalyzed by modification enzymes encoded in co‐regulated gene clusters [17]. Systematic investigation of RiPP antibacterial potential in endophytic fungi specifically remains limited relative to PKS, NRPS, and terpene pathways, reflecting both the recent development of bioinformatic RiPP detection tools and the technical challenges of characterizing heavily modified peptide products. Table 2 compiles representative antibacterial metabolites from each pathway family reported between 2018 and 2024, updated with mechanistic annotations.

**TABLE 2:** Representative antibacterial metabolites from endophytic fungi (2018–2024) with biosynthetic pathway and mechanism.

Compound	Pathway	Endophyte	Host plant	Activity	Mechanism	Ref.
Rasfonin	PKS	*Penicillium sp*.	*Ginkgo biloba*	MIC 10 µg/mL (S. aureus)	Membrane disruption	[38]
Subplenones A–J	PKS (xanthones)	*Subplenodomus sp*.	*Gentiana roots*	MIC 0.25 µg/mL (MRSA)	Topoisomerase inhibition (putative)	[19]
Parengyomarins A–B	PKS macrodiolide	*Parengyodontium album*	*Avicennia marina*	MIC 0.4 µM (S. aureus)	Membrane insertion	[20]
Beauvericin	NRPS‐PKS hybrid	*Fusarium oxysporum*	*Edgeworthia*	MIC 3.9 µM (S. aureus)	Ionophore; membrane potential dissipation	[26]
Fusaristatin A	PKS‐NRPS lipopeptide	*Fusarium* sp.	*Various*	Anti‐Xanthomonas	Membrane disruption (lipopeptide)	[36]
Cytochalasins	PKS‐NRPS macrocycle	*Phomopsis* sp.	*Camptotheca acuminata*	Anti‐Bacillus, anti‐Candida	FtsZ inhibition; actin disruption	[33]
Trichodermin	TPS (trichothecene)	*Trichoderma* sp.	*Various*	Anti‐MRSA (in vitro)	Peptidyl transferase inhibition (eukaryotic 60S); secondary mechanism vs. bacteria	[23]
Helvolic acid	TPS (fusidane)	*Aspergillus* sp.	*Various*	Antibacterial	EF‐G translocation block	[24]
Paclitaxel	TPS + phenylpropanoid	*Pestalotiopsis microspora*	*Taxus spp*.	Anticancer; antibacterial	Tubulin/FtsZ stabilization	[39]
Tricin	Phenylpropanoid	*Endophyte, Poa ampla*	*Grass*	Antioxidant; antibacterial	Membrane permeability	[40]
Resveratrol	Shikimate‐phenylpropanoid	*Alternaria* sp*. MG1*	*Grapevine*	Phytoalexin range	FtsZ inhibition; efflux pump inhibition	[29]

^*^Mechanisms labeled “putative” are inferred from structural analogy to known pharmacophores and have not been confirmed by direct biochemical assay for the specific compound listed.

## 4 Genomic and Metagenomic Approaches to Antibacterial Compound Discovery: Capabilities and Limitations

Whole‐genome sequencing, transcriptomics, metagenomics, and computational BGC mining have collectively transformed the pace at which biosynthetic capacity is mapped in endophytic fungi. However, each approach carries specific technical limitations that constrain the conclusions that can be drawn from its data, and a rigorous understanding of these limitations is essential for evaluating the reliability of genome‐mining‐derived predictions before committing experimental resources to their follow‐up.

### 4.1 Whole‐Genome Sequencing and Transcriptomics: Power and Blind Spots

Whole‐genome sequencing (WGS) using Illumina short‐read platforms has become the standard first step in endophytic BGC inventorying. Short‐read assemblies typically resolve 80%–95% of fungal genome content, but PKS and NRPS genes, which encode very large multidomain enzymes (often 10–30 kb per gene), frequently contain internal repetitive sequences that cause assembly fragmentation. A PKS gene split across two contigs by an assembly gap will be reported as two truncated, apparently non‐functional fragments rather than one intact biosynthetic enzyme, and antiSMASH will either miss the cluster entirely or report it as a partial cluster with uncertain product prediction [41, 42]. This systematically underestimates BGC content, particularly for the largest and most structurally complex cluster types. Long‐read sequencing platforms, specifically PacBio SMRT and Oxford Nanopore Technologies (ONT), resolve this problem by generating reads of 10–100 kb that span most repetitive regions in PKS and NRPS genes. Chromosome‐level assembly using Hi‐C chromatin proximity ligation further scaffolds contigs into complete chromosomes, ensuring that BGCs split across assembly gaps are recovered intact [41]. A long‐read assembly of *Aspergillus wentii* recently recovered terpene synthase genes absent from earlier short‐read drafts of the same genome [41], illustrating the practical impact of platform choice.

Transcriptomic profiling (RNA‐seq) identifies which BGCs are transcriptionally active under specific culture conditions. The leucinostatin BGC in *Purpureocillium lilacinum* was assigned to its metabolite product through combined expression profiling and targeted gene disruption [43]. RNA‐seq of Alternaria sp. MG1 identified co‐expressed phenylpropanoid pathway genes that collectively constitute the resveratrol biosynthesis route [29]. The critical limitation of transcriptomics for silent cluster discovery is circular: clusters that are genuinely silent produce no transcripts and therefore generate no signal in RNA‐seq data. Integrating RNA‐seq with ChIP‐seq profiling for repressive histone marks (H3K9me3, H3K27me3) offers a route toward identifying silenced clusters prospectively by their chromatin state rather than their transcriptional output, though such multi‐omic approaches remain technically demanding in non‐model fungi.

### 4.2 Metagenomics: Culture‐Independence and Its Inherent Constraints

Metagenomic sequencing of plant tissue DNA captures biosynthetic gene content from entire endophytic communities, including species that cannot be cultured under standard laboratory conditions. Targeted metagenomic surveys using degenerate PCR primers against conserved PKS ketosynthase, NRPS adenylation, and terpene synthase domains have confirmed biosynthetically active endophytic communities in plant tissues ranging from mangrove roots to tree bark, revealing biosynthetic diversity far exceeding what isolated strains from the same tissues would predict [44, 45]. Three practical constraints limit the depth and reliability of endophytic metagenomic data. First, plant genomic DNA typically constitutes 90%–99% of total DNA extracted from plant tissue, creating a severe imbalance that reduces effective sequencing depth for microbial sequences despite available depletion strategies. Second, metagenomic assembly of BGCs from complex communities generates chimeric contigs from closely related strains that can produce artificial BGC combinations not present in any real organism. Third, binning assembled contigs into draft genomes is unreliable for low‐abundance community members, which may include the most chemically novel endophytic species. These constraints mean that metagenomic BGC predictions require independent experimental validation before the predicted chemistry can be accepted as real.

### 4.3 Bioinformatics and Machine Learning: Prediction Accuracy and Scope Boundaries

antiSMASH 6.0 identifies BGCs by scanning genome sequences for combinations of biosynthetic domain signatures, classifies them by cluster type, and assigns similarity scores to known clusters in the MIBiG reference database [46]. Detection sensitivity is high for well‐characterized cluster types with canonical domain arrangements but substantially lower for atypical architectures and the plant‐like shikimate pathway clusters discussed in Section 3.5 that fall outside fungal training data. BiG‐SCAPE complements antiSMASH by grouping predicted BGCs across multiple genomes into gene cluster families (GCFs) based on Pfam domain content similarity, enabling comparative biosynthetic genomics across endophyte collections [46]. Seq2PKS represents a notable advance in polyketide structure prediction, but its operating principle is frequently mischaracterized: it is not a sequence‐to‐structure predictor that infers a compound structure from a BGC sequence alone. It is a paired genomics–metabolomics tool that uses both genome sequence data and tandem mass spectrometry (MS/MS) fragmentation spectra to propose candidate structures for detected metabolite ions [46]. It cannot predict products of silent clusters, as no metabolite MS/MS data exist for compounds not yet produced. Deep learning approaches to BGC bioactivity prediction [47] have shown promising results primarily in actinomycete systems; direct application to fungal endophytes is constrained by training data imbalance in MIBiG, where fungal BGC diversity is less uniformly sampled than bacterial. Molecular networking through GNPS groups MS/MS spectra by structural similarity, enabling dereplication and structural analogue identification [48, 49], but like Seq2PKS it requires compounds to be produced and detectable in culture. Table 3 summarizes the principal approaches, their capabilities, and their specific limitations.

**TABLE 3:** Genomic, transcriptomic, metagenomic, and computational tools for BGC discovery in endophytic fungi, with capabilities and limitations.

Tool / Approach	Principle	Documented capability	Key limitation in endophyte context	Example	Ref.
WGS (Illumina short‐read)	Shotgun sequencing + de novo assembly	Full BGC inventory; antiSMASH annotation; scalable and cost‐effective	Fragments large PKS/NRPS genes at internal repeats; systematically undercounts complex cluster types	*Fusarium* sp. VM‐40: 56 BGCs identified	[10]
WGS (PacBio/ONT long‐read) + Hi‐C	Long reads span repetitive regions; Hi‐C scaffolds chromosomes	Resolves complete PKS/NRPS gene structures; chromosome‐level assemblies improve BGC boundary accuracy	Higher cost; ONT homopolymer errors; limited endophyte genomes sequenced to this standard	*A. wentii* terpene synthase recovery	[41]
RNA‐seq (transcriptomics)	mRNA sequencing identifies expressed genes and co‐regulated clusters	Identifies active clusters; links gene expression to metabolite profiles; validates BGC boundaries	Silent clusters generate no signal; cannot detect cryptic pathways; requires condition‐matched controls	*Alternaria* sp. MG1 resveratrol pathway	[29]
Metagenomics (shotgun)	Total DNA sequencing from plant tissue	Captures uncultured endophyte BGC content; reveals community‐level biosynthetic diversity	Plant DNA dominates (90%–99%); chimeric assembly in complex communities; low‐abundance organisms underrepresented	PKS/NRPS diversity in Taxus endophyte communities	[45]
antiSMASH 6.0	Domain signature scanning + MIBiG comparison	Automated BGC detection, type classification, and similarity scoring; free and widely supported	Lower sensitivity for atypical domain orders, plant‐like shikimate clusters, and architectures outside training data	56 BGCs in *Fusarium* VM‐40	[46]
BiG‐SCAPE	Pfam domain network clustering across genomes	Groups BGCs into gene cluster families; enables comparative biosynthetic genomics across strains	Clustering thresholds are empirical; GCF boundaries do not always reflect chemical diversity	Pan‐genome BGC comparison in Aspergillus	[46]
Seq2PKS	Paired MS/MS + genome sequence polyketide structure prediction	Proposes candidate structures for detected polyketide ions by constraining structural search space using PKS domain logic	Requires detectable metabolite MS/MS data; cannot predict products of silent clusters; limited to type I cis‐AT PKS systems	Novel polyketide structure assignment	[46]
ML / deep learning BGC prediction	Supervised learning on annotated BGC datasets	Predicts bioactivity class from cluster features; prioritizes novel clusters for investigation	Models trained primarily on bacterial BGC data; lower accuracy for fungal clusters due to MIBiG training set bias	BGC bioactivity scoring in actinomycetes	[47]
GNPS molecular networking	MS/MS cosine similarity spectral grouping	Dereplicate known compounds; identify structural analogues; link metabolite ions to BGC predictions	Requires compound to be produced and detectable; no utility for silent clusters; lower spectral matching for truly novel scaffolds	Metabolomics–genomics integration in endophyte studies	[48, 49]

## 5 Strategies to Activate Silent Biosynthetic Gene Clusters and Enhance Metabolite Production

The majority of endophytic BGCs are transcriptionally silent under routine laboratory monoculture conditions, and converting this silent genetic potential into detectable chemical output is one of the central practical challenges in endophyte‐based drug discovery. Five principal experimental strategies have been developed: chemical elicitation, co‐cultivation, epigenetic modulation, the one strain many compounds (OSMAC) approach, and synthetic biology. Each strategy acts at a different level of the regulatory hierarchy: elicitors and co‐culture stimuli operate at the intracellular signaling level; epigenetic modifiers act at the chromatin and transcriptional levels; and synthetic biology interventions act directly on gene expression and pathway architecture.

### 5.1 Chemical Elicitation: Signaling Mechanisms and Scope of Action

Chemical elicitors activate secondary metabolite production in endophytic fungi by mimicking environmental signals encountered in host‐associated or competitive contexts. Methyl jasmonate (MeJA) and salicylic acid (SA), widely used as fungal secondary metabolism elicitors, are perceived in plants through the JAZ‐COI1 and NPR1 receptor systems respectively. Fungal genomes do not encode structural homologues of COI1 or NPR1, so MeJA and SA do not activate fungal BGCs through the same receptor systems they use in plants [50, 51]. In fungi, MeJA treatment elevates intracellular reactive oxygen species (ROS) levels and activates MAP kinase cascades, particularly the HOG (high‐osmolarity glycerol) pathway and the cell wall integrity (CWI) pathway, which alter the phosphorylation state of transcription factors regulating secondary metabolite genes [52, 53]. SA may act through alterations in membrane fluidity and fatty acid signaling that modulate lipid‐derived second messenger cascades. Chitooligosaccharides are recognized by chitin‐binding pattern recognition receptors at the fungal cell surface, activating defense signaling cascades that overlap with secondary metabolite induction pathways. Metal ions at sub‐toxic concentrations modulate secondary metabolism through cofactor availability and oxidative stress responses; their effects are metabolite‐class specific because different biosynthetic enzymes have different cofactor requirements [54]. Chemical elicitation has practical advantages of low cost and absence of genetic manipulation requirements, but dose–response is highly strain‐specific, and combining elicitor treatment with transcriptomic monitoring of BGC expression is essential for mechanistic attribution to specific clusters [53, 54].

### 5.2 Co‐Cultivation: Ecological Signals and the Chemistry of Microbial Competition

Laboratory co‐culture recreates functionally relevant inter‐organismal signals in a controlled setting. Bacterial cell wall fragments (peptidoglycan, lipopolysaccharide) are recognized by fungal pattern recognition receptors and activate stress‐response pathways; quorum‐sensing molecules from bacteria alter gene expression in neighboring fungi; and physical contact between species can trigger epigenetic remodeling through contact stress responses [55, 56]. The induction of new polyketides when endophytic *Chaetomium* sp. was co‐cultured with *Bacillus subtilis* [57] likely involves recognition of bacterial cell wall components and activation of fungal defense signaling. The *Aspergillus–Streptomyces* co‐culture system reported by Netzker et al. [56] demonstrated that physical proximity contributes to metabolite induction alongside diffusible chemical signals, with separation by a dialysis membrane partially attenuating the response. A significant methodological limitation is attribution: determining whether a new compound is produced by organism A, B, or through biotransformation requires isotope labeling or genetic knockouts in the producer organism. High‐throughput microfluidic co‐culture platforms with genetic reporter strains for individual BGC expression are beginning to address this limitation [12].

### 5.3 Epigenetic Modulation: Chromatin Architecture and Transcriptional De‐Repression

Silent BGCs in fungi are frequently embedded in genomic regions characterized by H3K9me3, a repressive histone mark associated with constitutive heterochromatin, and by reduced histone acetylation at H3K9, H3K14, and H4K16. The heterochromatin protein HepA (HP1 homologue) binds H3K9me3 marks and maintains chromatin compaction, creating a stable repressive state [13, 58]. DNA methyltransferase (DNMT) inhibitors such as 5‐azacytidine are incorporated into DNA as cytidine analogues during replication. When a DNMT encounters the modified base, it forms a covalent enzyme‐DNA adduct that traps the enzyme and prevents completion of the methylation reaction, leading to passive demethylation over subsequent cell divisions [13, 59]. Histone deacetylase (HDAC) inhibitors including vorinostat (SAHA) and nicotinamide chelate the catalytic zinc ion in the HDAC active site, blocking deacetylation and causing gradual accumulation of hyperacetylated histones that adopt a more open, transcriptionally permissive conformation [12, 13]. Treatment of *Eupenicillium* sp. LG41 with nicotinamide produced new decalin‐type polyketides absent from monoculture [60], and combined 5‐azacytidine and SAHA treatment of *Alternaria* sp. activated alternariol and related metabolites [59], confirming that these chromatin interventions unlock genuine new chemistry. The primary limitation is non‐specificity: global HDAC or DNMT inhibition affects the entire genome simultaneously and can co‐activate toxin‐producing or otherwise undesirable clusters alongside intended targets. Locus‐specific activation via dCas9 fused to histone acetyltransferase domains targeted by guide RNA to specific BGC promoters offers a path toward targeted chromatin remodeling without global effects, though this approach has not yet been systematically applied in endophytic species [58].

### 5.4 Synthetic Biology and Metabolic Engineering: Precision Activation and Heterologous Production

CRISPR‐Cas9 applied in *Lophiotrema* sp. F6932 demonstrated that targeted PKS gene disruption abolished palmarumycin and melanin production, directly confirming the PKS gene's role in both pathways and establishing gene function without ambiguity [61]. Regulatory gene manipulation offers a broadly applicable strategy: LaeA, an SAM‐binding protein component of the trimeric velvet complex (VeA–VelB–LaeA), coordinates the expression of numerous BGCs through effects on heterochromatin organization [15]. Its precise biochemical mechanism remains under investigation, but overexpression of LaeA homologues in *Aspergillus* species has consistently activated multiple previously silent BGCs. Overexpression of FnVeA in *Fusarium nematophilum* increased the diversity of detected secondary metabolites and elevated antitumor activity in culture extracts [14]. Heterologous expression of terpene synthase genes from a Helotiales sp. endophyte (strain BL73) in Streptomyces yielded linalool, an oxidized derivative, and a structurally novel terpenoid [62], demonstrating that the *Streptomyces chassis* can express endophytic terpene genes with appropriate precursor flux. Selection of host organism based on cofactor compatibility and precursor availability is a critical design decision: for NRPS clusters requiring phosphopantetheinylation of carrier protein domains, fungal hosts such as *Aspergillus niger* or engineered *Saccharomyces cerevisiae* are more appropriate than bacterial chassis.

### 5.5 Integrated Strategies: Mechanistic Rationale for Combining Approaches

OSMAC Approach: Culture Condition Variation as a Discovery Strategy. The one strain many compounds (OSMAC) approach exploits the observation that a single microbial strain produces dramatically different metabolite profiles when cultivated under varying conditions of nutrient composition, temperature, aeration, and culture duration. In endophytic fungi, OSMAC has demonstrated particular productivity: *Diaporthe kyushuensis* ZMU‐48‐1 produced diverse antifungal metabolites under altered cultivation conditions undetected under standard monoculture [63]. The mechanistic basis involves shifts in the basal transcription factor landscape driven by altered nutrient sensing through cAMP‐PKA and TOR signaling, modifying the activity of LaeA and the velvet complex. OSMAC requires no specialized equipment or transformation protocols and can be applied to any cultivable strain [54, 63].

No single activation strategy reliably unlocks all silent BGCs in a given endophytic fungus because different silent clusters are silenced through different regulatory mechanisms. A cluster repressed by H3K9me3 heterochromatin will not respond to MeJA treatment if its upstream signaling pathway does not connect to the HepA‐heterochromatin axis; a cluster requiring a specific transcription factor absent under monoculture will not respond to HDAC inhibition alone if that factor is not among the genes de‐repressed by histone hyperacetylation. The most productive documented combinations pair strategies acting on different regulatory levels: epigenetic priming (HDAC inhibition) followed by chemical elicitation (MeJA) opens chromatin at BGC loci and then provides a transcriptional activating signal, mimicking the sequential host entry and competitive signaling that might activate an endophyte's defense chemistry in planta. Combined OSMAC media variation with epigenetic modifiers similarly pairs shifted transcription factor landscape with chromatin accessibility, as demonstrated in *Diaporthe kyushuensis* ZMU‐48‐1 where combined strategies unveiled diverse antifungal metabolites undetected under standard conditions [63]. A predictive framework matching activation strategy to silencing mechanism, using ChIP‐seq profiling to map BGC chromatin state, would move this field from empirical screening toward rational strategy selection. Table 4 summarizes the five main strategies with molecular mechanisms and limitations. Figure 2 illustrates the three regulatory levels controlling BGC expression in endophytic fungi and the experimental strategies that target each level.

**TABLE 4:** Strategies for activating silent BGCs and enhancing antibacterial metabolite production in endophytic fungi.

Strategy	Molecular mechanism	Representative outcome	Current limitation	Refs.
Chemical elicitation (MeJA, SA, chitooligosaccharides, metal ions)	Activates MAP kinase stress cascades (HOG, CWI) and ROS signaling; alters phosphorylation of secondary metabolism transcription factors. Does NOT act through plant JAZ‐COI1 or NPR1 receptors.	MeJA and SA elevated antibiotic titers several‐fold in fungal fermentations; ABA increased sesquiterpene output in *Artemisia* endophytes; metal ions shifted carbon flux toward polyketide biosynthesis	Dose‐response highly strain‐specific; off‐target growth inhibition at supra‐optimal doses; mechanistic attribution to specific BGCs requires concurrent RNA‐seq monitoring	[50, 51, 52, 53, 54]
Co‐cultivation (with bacteria, competing fungi, or plant tissue)	Bacterial cell wall components (peptidoglycan, LPS) activate fungal pattern recognition; quorum‐sensing signals and contact stress trigger chromatin remodeling; physical interaction adds contact‐mediated signaling	*Chaetomium* sp. + B. subtilis yielded new polyketides absent from monoculture; *Aspergillus–Streptomyces* co‐culture induced metabolites undetected in pure cultures	Metabolite attribution between organisms requires genetic knockouts or isotope labeling; outcomes variable across laboratory conditions; industrial scale‐up is technically complex	[55, 56, 57]
Epigenetic modulation (5‐azacytidine, SAHA, nicotinamide, valproic acid)	5‐azacytidine: DNMT inhibition by covalent enzyme trapping; passive demethylation over cell divisions. SAHA/nicotinamide: HDAC inhibition via Zn2+ chelation; histone hyperacetylation; chromatin relaxation at heterochromatic BGC loci	5‐azacytidine + SAHA activated alternariol and altertoxin II in *Alternaria* sp.; nicotinamide induced novel decalin polyketides in *Eupenicillium* sp. LG41; *Bartalinia* sp. produced new peptahivin after combined treatment	Global DNMT/HDAC inhibition activates all responsive loci simultaneously; toxigenic clusters may be co‐activated; locus‐specific activation via dCas9‐HAT not yet applied in endophytic fungi	[58, 59, 60]
Synthetic biology (CRISPR‐Cas9, regulatory overexpression, heterologous expression)	CRISPR: targeted gene disruption or promoter editing for BGC confirmation and activation. Regulator OE: LaeA, VeA influence heterochromatin organization globally. Heterologous expression: BGC reconstitution in tractable host with appropriate precursor flux	CRISPR disruption confirmed palmarumycin PKS gene in Lophiotrema sp.; FnVeA OE increased antitumor metabolite diversity in *F. nematophilum*; Helotiales BL73 terpene synthase expressed in Streptomyces yielded novel terpenoid	Transformation efficiency low in many endophytic fungi; stable integration not always achieved; host selection for heterologous expression requires matching cofactor and precursor requirements	[61, 62, 63, 64, 65, 66]

**FIGURE 2:**
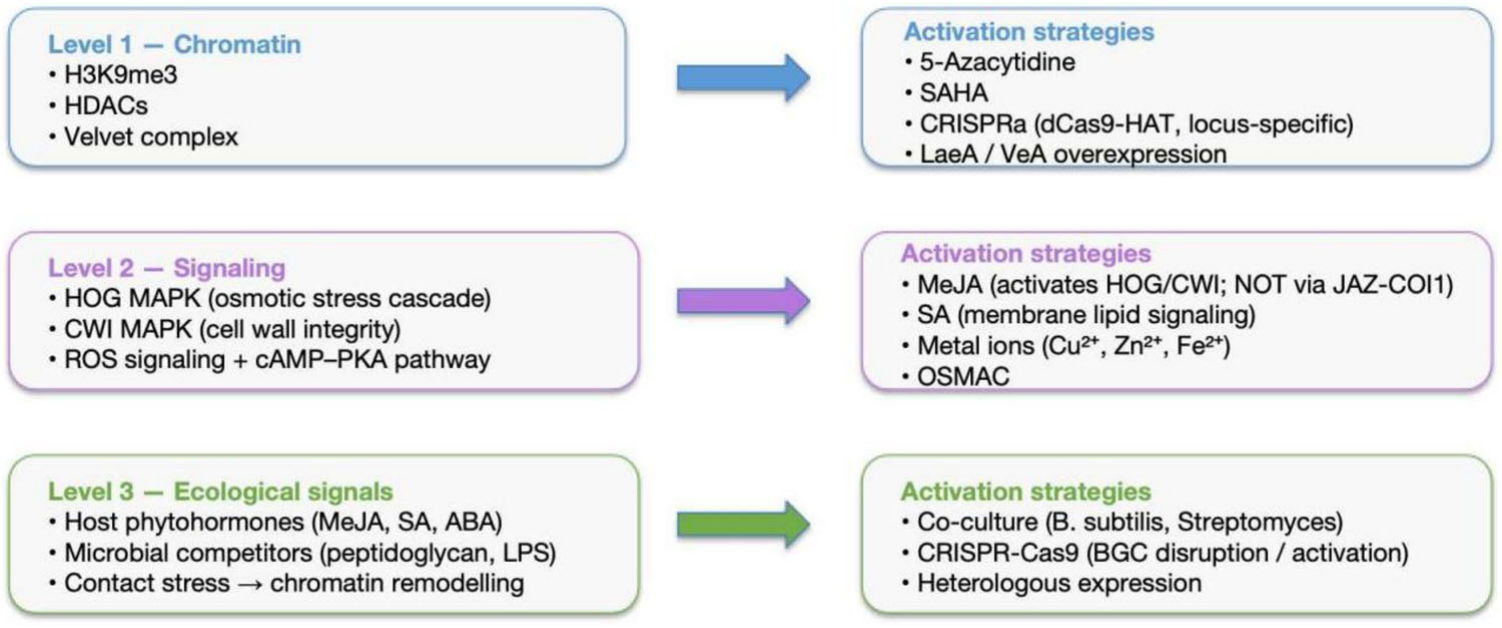
BGC activation regulatory framework in endophytic fungi.

## 6 Applications in Drug Discovery: Concrete Progress and Translation Barriers

Endophytic fungi have generated antibacterial leads across the full spectrum of drug discovery stages, from hit identification to mechanism characterization to preliminary in vivo evaluation. This section examines that progress with precision, distinguishing what has been demonstrated from what remains aspirational.

### 6.1 Compounds With Characterized Mechanisms and Demonstrated Potency

The most clinically relevant finding from endophytic antibacterial research is not a single potent compound but a class of structurally diverse resistance‐modifying agents that address MRSA through PBP2a inhibition. Spiroaspertrione A and aspermerodione from *Aspergillus* sp. TJ23 [16, 31] reduce the oxacillin MIC against MRSA to clinically achievable concentrations when used in combination, demonstrating proof of concept that endophytic chemistry can reverse a clinically significant resistance mechanism. The structural basis of this activity, occupancy of the PBP2a allosteric site that triggers the conformational change required for active‐site opening to beta‐lactams, has been characterized crystallographically for related compounds and provides a foundation for rational analogue design.

Trichodermin and related trichothecene sesquiterpenes from Trichoderma endophytes inhibit eukaryotic ribosomal peptidyl transferase activity by binding within the A‐site of the 60S ribosomal subunit [64, 65]. In vitro anti‐MRSA activity has been documented [23]; the molecular basis of antibacterial activity is distinct from eukaryotic ribosome inhibition since prokaryotic 70S ribosomes differ structurally at the peptidyl transferase center, and likely involves a secondary mechanism. Overexpression of the tri5 gene in *T. brevicompactum* increased trichodermin production to levels sufficient for preclinical evaluation [65], demonstrating that yield limitations can be addressed through targeted pathway engineering. The most potent single compound identified through epigenetic activation, the most active decalin‐type polyketide analogue from *Eupenicillium* sp. LG41, exhibited an MIC as low as 0.1 µg/mL against *S. aureus* under broth microdilution conditions [60], comparable to vancomycin and superior to many approved antibiotics against the same organism. That this compound was absent from monoculture metabolite profiles of the same strain makes it one of the clearest demonstrations in the endophyte literature that epigenetic activation directly enables discovery of highly potent compounds that conventional approaches miss entirely. Beyond these lead series, several additional endophytic compounds with well‐characterized mechanisms merit mention. Helvolic acid, a fusidane‐type triterpene from *Aspergillus* endophytes, recapitulates the mechanism of the clinically approved antibiotic fusidic acid by trapping the EF‐G:GDP:ribosome ternary complex in the post‐translocational state [24]. Beauvericin from Fusarium oxysporum endophytes functions as a cation‐selective ionophore with documented anti‐MRSA activity (MIC 3.9 µg/mL) [26]; its ionophore mechanism, dissipating membrane potential rather than targeting a specific protein, is inherently resistant to single‐target resistance development. Preussolides A and B from *Preussia typharum* endophytes exhibit selective inhibition of *Cryptococcus neoformans* through macrolide membrane‐insertion mechanisms [65], extending the range of endophytic antibacterial leads to opportunistic fungal pathogens of significant clinical concern.

### 6.2 Critical Evaluation of Bioactivity Data Quality

A substantive limitation of the endophytic antibacterial literature is the heterogeneity of bioactivity testing methodology across studies. MIC values in Tables 1 and 2 were derived from studies using broth microdilution (BMD), agar dilution, disk diffusion, and microplate colorimetric assays. These methods are not directly comparable: BMD per CLSI and EUCAST guidelines uses defined inocula, medium, and incubation conditions, and its MIC values are clinically interpretable against resistance breakpoints. Disk diffusion inhibition zones and colorimetric IC_50_ values measured in DMSO‐containing media at non‐standardized inocula are not. The field would benefit from greater adherence to CLSI M27, M38, or M07 guidelines in primary screening, and from routine reporting of assay conditions, inoculum size, medium composition, and DMSO concentration. Until standardization improves, MIC comparisons across studies should be interpreted as indicative of approximate potency class rather than precise quantitative benchmarks.

### 6.3 The Translation Gap: From Hit to Clinical Candidate

No endophytic fungal metabolite has yet entered clinical trials as an antibacterial agent. Most endophytic antibacterial leads have been characterized only by MIC determination against a small organism panel under in vitro conditions, with no cytotoxicity selectivity index, no in vivo efficacy data in animal infection models, no pharmacokinetic profiling, and no stability assessment. The transition from an interesting MIC to a preclinical candidate requires all of these, and their absence reflects the limited resources available in academic natural product research groups rather than any inherent limitation of the compounds themselves.

The most realistic near‐term translational applications for endophytic chemistry are combination strategies rather than standalone new antibiotics. Resistance‐modifying agents such as spiroaspertrione A, which restore beta‐lactam efficacy against MRSA without requiring independent bactericidal potency, have a lower bar for clinical utility because they can be co‐dosed with existing approved drugs. Efflux pump inhibitors with the structural profile of resveratrol analogues represent a similar opportunity. Pursuing these combination strategies as the primary translational objective, rather than awaiting a standalone bactericidal lead to advance through a full development program, is the most evidence‐based path from the current state of endophytic antibacterial research toward clinical impact.

## 7 Challenges, Open Questions, and a Prioritized Research Framework

The challenges facing endophytic antibacterial research are not uniformly distributed in their difficulty or tractability. This section identifies the four most consequential bottlenecks, explains the specific experimental or methodological work that would resolve each one, and proposes an order of priority based on the degree to which each bottleneck constrains downstream progress.

### 7.1 Priority 1: BGC‐to‐Metabolite Linkage in Silent Clusters

The highest‐priority unresolved problem in the field is establishing reliable, scalable methods for linking silent BGC sequences to their encoded metabolite products. antiSMASH predicts 30–80 BGCs per endophytic fungal genome, of which fewer than 10%–20% correspond to identifiable metabolites under standard culture conditions. The remaining 80%–90% represent an uncharacterized chemical space whose scale dwarfs current output from the field. The most direct experimental path toward resolving this bottleneck combines three approaches in sequence. First, ChIP‐seq using antibodies against H3K9me3 and H3K27me3 marks would map which specific BGCs are embedded in repressive heterochromatin, enabling targeted epigenetic activation using locus‐specific dCas9‐HAT CRISPRa rather than global HDAC inhibition. Second, for clusters that remain silent after epigenetic activation, heterologous expression in a characterized host, either *Aspergillus niger* for fungal PKS and NRP clusters or Streptomyces coelicolor M1152 for terpene synthase genes, would allow encoded chemistry to be expressed independently of native regulatory constraints. Third, paired genomics‐metabolomics analysis applied to the heterologous expression strains would then assign MS/MS metabolite ions to the expressed BGC. The bottleneck in implementing this pipeline at scale is transformation efficiency in endophytic fungi; developing more broadly applicable transformation protocols, or establishing phylogenetically representative endophytic chassis strains with robust transformation systems, would represent an infrastructure investment with disproportionate field‐wide returns.

### 7.2 Priority 2: Metabolite Yield Stability and Sub‐Culturing Decline

The progressive loss of secondary metabolite productivity during serial sub‐culturing is a practical barrier that makes endophytic fungi unreliable as production platforms even when active compounds are identified. The likely mechanism is epigenetic drift: in the absence of host‐derived signals that maintain the native chromatin state in planta, heterochromatic silencing of BGC loci accumulates with each cell division [66]. Maintaining cryopreserved stocks from primary isolation and returning to early‐passage material for production runs limits but does not eliminate the problem. A more fundamental solution requires either constitutive expression of LaeA to maintain the chromatin state supporting BGC expression, or stable CRISPRa constructs that continuously maintain the active chromatin state at target loci across passages. A recent study of *Diaporthe kyushuensis* ZMU‐48‐1 demonstrated that systematic culture condition variation can partially rescue productivity in strains showing yield decline [63], suggesting that metabolic dormancy rather than irreversible silencing is the mechanism in at least some cases, which is an encouraging finding for recovery strategies.

### 7.3 Priority 3: Genome Assembly Quality and Annotation Completeness

The endophytic mycology community has largely continued to publish short‐read draft genome assemblies, which systematically miss large PKS and NRPS genes as described in Section 4.1. A systematic effort to re‐sequence previously published endophyte draft genomes using long‐read platforms would recover substantial numbers of BGCs currently missing from the literature, with direct consequences for how accurately the field estimates total chemical potential. Annotation completeness is a related but distinct problem: automated annotation tools trained primarily on model fungal genomes systematically misannotate genes in phylogenetically distant endophytic species, particularly in the *Xylariales, Diaporthales*, and *Helotiales*. Establishing community‐curated BGC databases specific to endophytic fungi, analogous to MIBiG but with endophyte‐specific taxonomic and ecological metadata, would accelerate annotation accuracy across the field.

### 7.4 Priority 4: Translational Biology and Preclinical Development

The absence of any endophytic antibacterial compound in clinical development reflects a genuine gap in preclinical biology. For resistance‐modifying meroterpenoids (spiroaspertrione A, aspermerodione), the priority experiments are: (1) determination of the minimum potentiating concentration (MPC) in combination with oxacillin against a panel of clinically derived MRSA isolates with characterized mecA genotypes; (2) mammalian cell cytotoxicity profiling against HEK293 and HepG2 cell lines to establish a selectivity index; (3) in vivo efficacy in a murine thigh infection model with MRSA using spiroaspertrione A plus sub‐therapeutic oxacillin. These experiments can be performed on the natural product directly and would collectively constitute a data package sufficient to support a translational grant application. For the most potent endophytic polyketides, the immediate priority is semi‐synthesis of the most active natural analogue to produce quantities sufficient for in vivo work, combined with a minimal SAR survey of 5–10 analogues to identify which structural features are dispensable, which are required for potency, and which are responsible for any observed cytotoxicity.

Regulatory and ethical considerations are directly relevant to the translation pathway and deserve mention beyond the brief acknowledgment they typically receive. The use of CRISPR‐modified or otherwise genetically engineered endophytic strains for compound production is subject to GMO regulations in most jurisdictions, affecting production‐site requirements and the regulatory pathway for the derived natural product. Intellectual property constraints are most significant for production processes and semi‐synthetic derivatives of compounds in active patent families, rather than for the natural products themselves, which in most cases cannot be patented under current IP frameworks. Early regulatory engagement with agencies such as the FDA or EMA for compounds approaching preclinical development status is a practical recommendation that is routinely absent from natural product reviews but is directly relevant to translational success.

## 8 Conclusion

This review has examined endophytic fungi as antibacterial compound sources across three connected dimensions: the structural chemistry and mechanisms of action of characterized metabolites, the biosynthetic pathways and regulatory architecture that generate and control that chemistry, and the experimental strategies available for activating silent genetic potential and improving metabolite yields. Several specific conclusions follow from this analysis.

The chemical case for endophytic fungi as antibacterial sources is stronger than the raw MIC literature suggests, because the most significant compounds are not simply potent inhibitors but mechanistically differentiated leads that address validated resistance mechanisms. Spiroaspertrione A and aspermerodione restore beta‐lactam efficacy against MRSA by engaging PBP2a, the resistance determinant responsible for the clinical failure of the most widely used antibiotic class. Resveratrol‐class stilbenes inhibit NorA‐family efflux pumps and FtsZ polymerization through mechanisms with no established clinical parallel. Fusidane‐type triterpenes produced independently by endophytic fungi recapitulate the target engagement of a clinically approved drug class, demonstrating that endophytic biosynthesis has independently arrived at antibacterial solutions that medicinal chemistry validated decades ago. These mechanistic observations, not the abundance of MIC reports in isolation, constitute the scientific argument for continued investment in endophytic antibacterial research.

The biosynthetic argument is equally specific. Genome mining data consistently show that 80%–90% of endophytic BGCs are silent under standard culture conditions, and the activation strategies reviewed in Section 5 have demonstrated that this silence is conditional, not irreversible. The mechanistic basis for silence, involving H3K9me3 heterochromatin maintained by HepA and regulated by the velvet complex, is tractable through both pharmacological and genetic interventions whose molecular mechanisms are now sufficiently understood to guide rational strategy selection. The convergence of long‐read genome sequencing, locus‐specific CRISPRa tools, and paired genomics‐metabolomics platforms creates a toolkit that directly addresses the BGC‐to‐metabolite linkage problem identified throughout this review as the field's primary bottleneck.

Three open questions remain that define the most important research priorities for the next five years. First, whether plant‐like shikimate pathway genes in endophytic fungi reflect horizontal gene transfer, convergent evolution, or systematic laboratory contamination requires a rigorous phylogenomic analysis of shikimate pathway gene tree across plant‐endophyte pairs, combined with chromosomal localization data from long‐read assemblies. Second, whether the sub‐culturing productivity decline is epigenetic drift or irreversible genomic change has not been distinguished experimentally for most affected strains; ChIP‐seq profiling across early and late passage cultures would resolve this directly and determine whether recovery strategies are likely to succeed. Third, the preclinical efficacy of the most potent endophytic leads in relevant in vivo infection models remains completely uncharacterized; this is the most directly actionable gap and requires only the prioritization decisions and cross‐disciplinary collaborations described in Section 7.4.

The structural barriers to translating endophytic chemistry into clinical candidates, including transformation efficiency limitations, the absence of medicinal chemistry collaborators in most endophyte research groups, and the under‐representation of endophytic BGC diversity in ML training datasets, are solvable through community‐level coordination. Depositing validated BGC‐to‐metabolite links in MIBiG, standardizing bioactivity testing against CLSI guidelines, establishing representative endophytic chassis strains with robust transformation systems, and systematically re‐sequencing published draft genomes with long‐read platforms are investments whose returns would be shared across the entire field. The endophytic antibacterial research community is at a point where coordinated infrastructure development would have a larger impact on scientific output than any individual discovery program. Acting on that observation is the most consequential recommendation this review can offer.

## Author Contributions

**F.E., A.S.S., and A.X.G**. contributed to conceptualization, literature review, data interpretation, and manuscript writing and editing. **F.E**. served as corresponding author. **L.K., D.K., and K.M**. contributed to literature review, data collection, and organization of scientific materials. **W.M.N.H.W.S. and S.A**. contributed to critical revision of the manuscript, scientific guidance, and manuscript review. **K.W.K.T**. contributed to conceptualization, supervision, critical revision of the manuscript, and correspondence.

## Funding

This research was funded by the Internal Research Grant of New Uzbekistan University (grant number NewUU‐RP‐2025‐12); the Agency for Innovative Development of Uzbekistan (grant number FL‐11525098811); the Hong Kong Innovation and Technology Commission (grant numbers PRP/096/24FX, ITCPD/17‐9, and ITC‐CNERC14SC01); the Laboratory for Synthetic Chemistry and Chemical Biology under the Health@InnoHK Program and the State Key Laboratory of Synthetic Chemistry; the Chinese Medicine Development Fund (grant number 24B1‐2/053A_R1); and the Shenzhen Science and Technology Innovation Committee (grant number ZDSYS201707281432317).

## Conflicts of Interest

The authors declare no conflicts of interest.

## Data Availability

Data sharing not applicable to this article as no datasets were generated or analyzed during the current study.
